# Unilateral synchronous papillary renal neoplasm with reverse polarity and clear cell renal cell carcinoma: a case report with *KRAS* and *PIK3CA* mutations

**DOI:** 10.1186/s13000-020-01042-7

**Published:** 2020-10-06

**Authors:** Hyun Jung Lee, Dong Hoon Shin, Joon Young Park, So Young Kim, Chung Su Hwang, Jung Hee Lee, Jee Yeon Kim, Mee Young Sol, Jong Kil Nam

**Affiliations:** 1 Department of Pathology, School of Medicine, Pusan National University, Yangsan, Korea; 2 Research Institute for Convergence of Biomedical Science and Technology, Pusan National University Yangsan Hospital, Yangsan, Korea; 3 Department of Urology, School of Medicine, Pusan National University, Yangsan, Korea

**Keywords:** Papillary renal neoplasm with reverse polarity, Clear cell renal cell carcinoma, Multiple masses, *KRAS* mutation, *PIK3CA* mutation

## Abstract

**Background:**

The presence of histologically different neoplasms in the same organ is rare in pathologic practice. We report the first case of synchronous clear cell renal cell carcinoma (clear cell RCC) and papillary renal neoplasm with reverse polarity (PRNRP) with comprehensive immunohistochemical and molecular characterization using next-generation sequencing (NGS).

**Case presentation:**

A 61-year-old man was incidentally found to have a left renal mass on imaging studies performed for workup of left back pain and urine color change for 1 week. A laparoscopic left radical nephrectomy was performed. Gross examination showed lobulated masses measuring 5.6 × 4.0 × 3.3 cm in the upper to mid pole and 1.1 × 1.0 × 1.0 cm in the lower pole. Microscopic findings revealed these to be two different separate masses of clear cell renal cell carcinoma and papillary renal neoplasm with reverse polarity. NGS analyses revealed *KRAS* gene mutation (c.35G > T/p.G12V in exon 2) in the papillary renal neoplasm with reverse polarity, with *PIK3CA* gene mutation restricted to the clear cell renal cell carcinoma (c.1624G > A/p.E542K in exon 10).

**Conclusions:**

We report here an extraordinarily rare case of synchronous renal tumors of papillary renal neoplasm with reverse polarity and clear cell renal cell carcinoma. We identified simultaneous *KRAS* and *PIK3CA* mutations in two different renal masses in the same kidney for the first time. New pathologic assessment with comparative molecular analysis of mutational profiles may be helpful for tumor studies.

## Introduction

Renal cell carcinoma (RCC) is the most common solid tumor of the kidney and accounts for 2–3% of all malignancies in adults [1]. The most common subtypes of RCC are clear cell, papillary, and chromophobe RCC, and they account for approximately 75, 10, and 5% of cases, respectively [2]. Since 1997, papillary renal cell carcinoma has been classified into types 1 and 2 based on the morphologic findings [3]. Histologically, papillae are lined by cuboidal cells with scant basophilic cytoplasm on the fibrovascular cores in type I, whose nuclei are usually arranged in a single layer with a low International Society of Urological Pathology (ISUP) grade. In contrast, type II is defined by nuclear pseudostratification, higher ISUP grades, and abundant eosinophilic cytoplasm. Type I has a better prognosis than type II. Most recently, 18 cases of papillary renal neoplasm with reverse polarity (PRNRP) were described by Al-Obaidy et al. [4]. PRNRP is histologically characterized by thin branching papillae, or rarely, predominant tubules covered by bland oncocytic cells with apical low-ISUP-grade nuclei.

There are a few studies in the literature describing bilateral synchronous malignant renal tumors [5, 6] and coexisting benign and malignant tumors in the same kidney [7]. However, clear cell RCC and PRNRP arising within the same kidney have not yet been reported in the literature. The presence of histologically different neoplasms in the same organ is rare in pathologic practice. Herein, we present the first well-described case of synchronous PRNRP and clear cell RCC with immunohistochemical and NGS analysis. We also reviewed multiple synchronous renal masses in Pusan National University Yangsan Hospital between March 2010 and January 2020.

## Materials and methods

Paraffin sections were prepared (2-μm thickness) and stained using routine methods on VENTANA (Roche, Basel, Switzerland) and BOND-MAX (Leica Biosystems, Buffalo Grove, IL) autostainers with the following antibodies: AMACR (13H4, α-methylacylcoenzyme A racemase; Agilent Technologies, Santa Clara, CA), CD10 (clone 56C6, Leica Biosystems), cytokeratin 7 (CK7; clone OV-TL 12/30, Leica Biosystems), high-molecular-weight (HMW) keratin (clone 34βE12, Agilent Technologies), TFE3 (MRQ-37, Cell Marque; Rocklin, CA), E-cadherin (clone 4A2C7; Invitrogen, Carlsbad, CA), CAIX (clone ab15086; Abcam, Cambridge, United Kingdom), GATA3 (clone L50–823, Ventana, Tucson, AZ), vimentin (clone V9, Invitrogen, Camarillo, CA), and EMA (clone E29, DAKO, Carpinteria, CA). Stained slides were scored for the presence and distribution of positive immunostaining.

DNA libraries were prepared using the NGeneBio library prep kit (NGeneBio, Seoul, Korea), enriched for 120-kb fragments, and sequenced with a paired-end 300-bp (150 bp each) protocol using a MiSeq platform (Illumina, San Diego, CA). Targeted sequencing raw data were obtained in FASTAQ format. Raw reads were aligned against the reference human genome assembly (GRCh37/hg19) using NGeneAnalysis v1.4.4.0 software (NGeneBio). A minimum coverage of 20 reads per base-pair was subsequently used for variant calling. Variants with a variant allele frequency of less than 5% were excluded. Variants outside 10 bases from exon-intron boundaries were also excluded from analysis.

## Case presentation

A 61-year-old man was incidentally found to have a left renal mass on imaging studies performed for workup of left back pain and urine color change for 1 week. A computed tomographic imaging scan of the kidneys revealed an enhancing mass with central necrosis and a sub-centimeter-sized hypodense nodule. Laparoscopic left radical nephrectomy was performed.

**Fig. 1:**
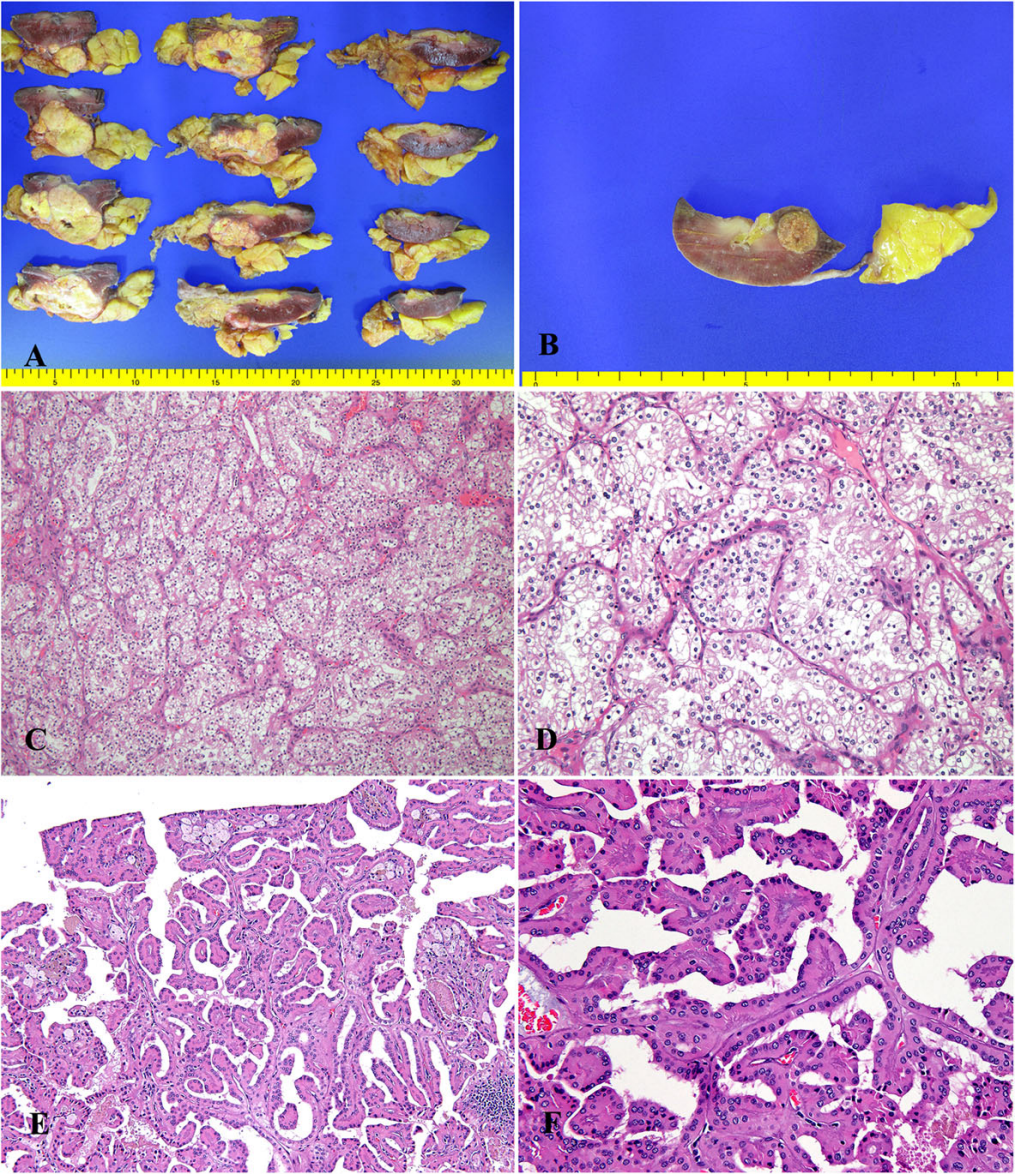
The cut surface of the mass was bright golden yellow with areas of gray-white fibrosis and hemorrhage (**a**). A separate small nodule was present in the lower pole, measuring 1.2 × 1.0 × 0.6 cm (**b**). Microscopic findings of clear cell renal cell carcinoma (**c**). High-power view of clear cell renal cell carcinoma (**d**). Microscopic findings of papillary renal neoplasm with reverse polarity (PRNRP) (**e**). High-power view of PRNRP (**f**)

**Fig. 2:**
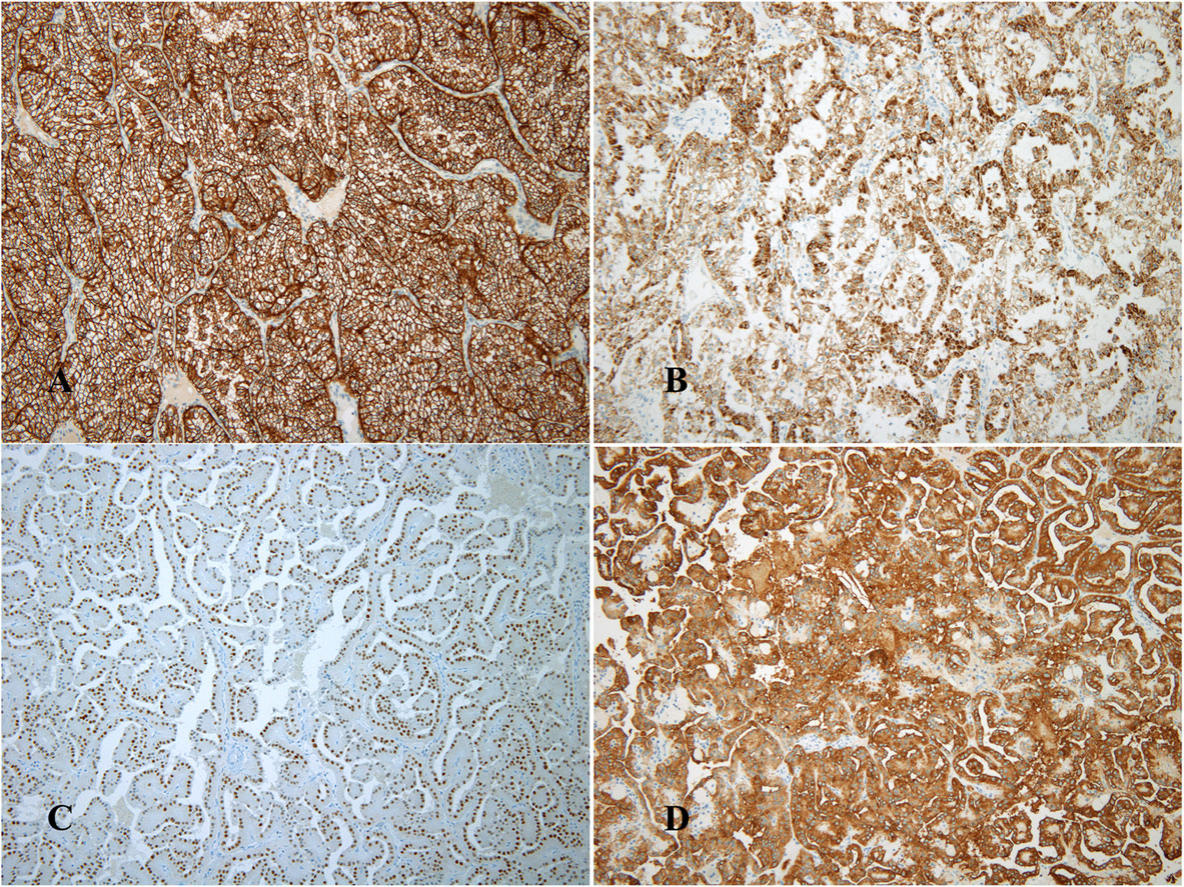
Immunohistochemical stain for CAIX in clear cell renal cell carcinoma shows membranous positivity (**a**). AMACR shows cytoplasmic positivity in clear cell renal cell carcinoma (**b**). GATA3 in papillary renal neoplasm with reverse polarity (PRNRP) shows strong nuclear positivity (**c**). CK7 shows diffuse cytoplasmic positivity in PRNRP (**d**)

Pathologic gross examination showed a vaguely circumscribed lobulated mass in the upper to mid pole, which measured 5.6 × 4.0 × 3.3 cm. The cut surface of the mass was bright golden yellow with areas of gray-white fibrosis and hemorrhage (Fig. 1a). Apart from this main mass, a separate small nodule was present in the lower pole, which measured 1.2 × 1.0 × 0.6 cm (Fig. 1b). Both tumors were confined to the kidney, having no extension into the perinephric adipose tissue, renal sinus, renal pelvis, or Gerota fascia. Hematoxylin and eosin (H&E)-stained sections from the larger tumor showed a well-circumscribed neoplasm composed predominantly of solid nests and lobules of large round cells with relatively monotonous round nuclei showing no mitoses and abundant granular eosinophilic cytoplasm (Fig. 1c and d). The second nodule was well-encapsulated, showing proliferation of delicate fibrovascular cores and variable numbers of macrophages. The papillary fibrovascular cores were thick and hyalinized. The tumors were cuboidal with eosinophilic cytoplasm. The nuclei were low-ISUP-grade nuclei and were arranged linearly and inverted toward the apical surface (Fig. 1e and f). For further evaluation, immunohistochemical stains were performed. The tumor cells were positive for CD10, AMACR, CAIX, and vimentin and negative for CK7, TFE3, E-cadherin, HMW keratin and GATA3. The separate small mass was positive for CK7, GATA3, AMACR, E-cadherin, HMW keratin, and EMA and negative for CD10, vimentin, CAIX, and TFE3 (Fig. 2). AMACR was moderately positive showing blush-like staining, which was weaker than that seen in the proximal renal tubules in the separate small mass. Based on the morphology and immunohistochemical findings, a chromophobe or translocation RCC were excluded. The findings support clear cell RCC and PRNRP. The tumor in the upper pole was clear cell RCC, with pathologic stage pT1b N0 M0. The histologic grade (World Health Organization [WHO]/ISUP nuclear grade) was 3. The second tumor in the lower pole was PRNRP, its pathologic stage was pT1a N0 M0, and its histologic grade (WHO/ISUP nuclear grade) was 2. Sarcomatoid features and necrosis were absent. The patient has been alive and well for 8 months after surgery.

**Fig. 3:**
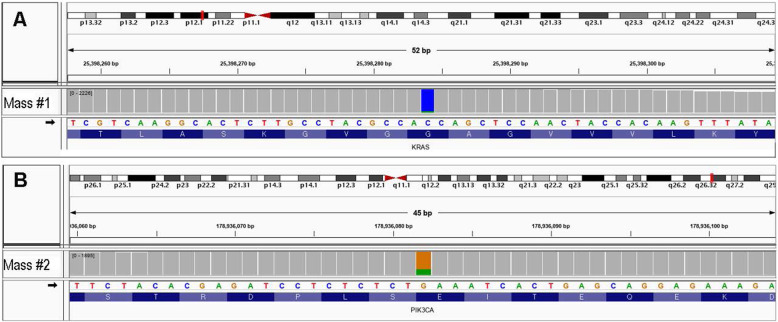
Integrative Genomics Viewer (IGV) snapshot of human *KRAS* gene location with missense mutation identified in papillary renal neoplasm with reverse polarity. The mutation is clustered in p.Gly12Val (**a**). IGV snapshot of human *PIK3CA* gene location with missense mutation identified in clear cell renal cell carcinoma. The mutation is clustered in p.Glu542Lys (**b**)

NGS analysis detected a *KRAS* mutation in the PRNRP in the exon 2-codon 12 junction (c.35 G > T resulting in p.G12V). A *PIK3CA* mutation was detected in the clear cell RCC in the exon 10-codon 542 junction (c.1624 G > A resulting in p.E542K) (Fig. 3).

## Discussion

Although multiple synchronous renal tumors are rare, the incidence of sporadic multifocal renal tumors at the time of diagnosis as reported in the literature is 4–20% [8–10]. This implies that observing multiple tumors in the kidney is relatively common. This is the first case to present synchronous clear cell RCC and PRNRP in the same kidney. PRNRP was first described by Al-Obaidy et al. in 2019 [4]. This study identified a PRNRP with a *KRAS* mutation and a co-occurring clear cell RCC with a *PIK3CA* mutation for the first time in the literature. The different mutation results of NGS analysis supports the biologically different diagnoses of these two synchronous renal masses.

*KRAS* mutations are frequently found in adenocarcinomas of the lung, colon, and pancreas [11, 12]. Varied papillary lesions, including intraductal papillary mucinous neoplasms of the pancreas and urothelial papillomas of the bladder, also have frequent *KRAS* mutations [13, 14]. *KRAS* mutations are rare events in kidney tumors, however, it has recently been found to be a characteristic feature of PRNRP as reported by multiple studies, including the current reported cases [15–17].

*PIK3CA* mutations are rare in clear cell RCC and are present only in 2–5% of tumors [18, 19]. *PIK3CA* codes for the catalytic subunit of phosphoinositide-3-kinase (PIK3), a key enzyme of the mTOR pathway; therefore, the mTOR inhibitor, everolimus, may be effective for this type of mutation.

The most common symptoms reported in RCC are hematuria in 90% of cases, flank pain in 19%, and mass effect in 14% [20]. Our patient presented with the first two symptoms. Regarding aggressiveness, clear cell RCC presents the greatest malignant potential and a 5-year survival rate of 70%, while papillary and chromophobe RCCs are associated with less metastatic potential and an overall 5-year survival of 88 and 94% [21].

Radical nephrectomy is considered the standard procedure for treating malignant renal tumors. However, recent studies show that patients with sporadic single or multiple ipsilateral renal tumors may undergo nephron-sparing surgery, with oncologically comparable results with low morbidity and recurrence rates.

Awareness of the coexistence of multiple synchronous tumors of different pathologic neoplasms in the same kidney is important for managing such cases, and nephron-sparing surgery or active surveillance may be warranted for some renal masses [22]. The fact that the pathological concordance rate is as low as 67.3% and the grade concordance rate is 62.5% [23] suggests that if a biopsy is indicated preoperatively, each nodule should be biopsied for diagnosis [8]. Different tumors will have different prognoses and degrees of aggressiveness.

There were seven cases of multiple kidney masses in Pusan National University Yangsan Hospital from March 2010 to January 2020, and Table 1 shows the characteristics of these ipsilateral multiple renal masses according to subtype. While five cases involved multiple clear cell RCCs, one presented papillary and clear cell RCCs, whereas the other had papillary RCC with metanephric adenoma. Two reports of large numbers of patients concluded that 5–6% of multiple ipsilateral renal tumors develop a contralateral metachronous recurrence and this risk is 5 times that of patients with a sporadic single tumor [23, 24]. We suggest that multiple ipsilateral synchronous RCCs of different histologic subtypes need to be followed closely and operations for each mass are necessary. The frequency of clinical multifocality is consistent with reported local recurrence rates following partial nephrectomy. The impact of tumor multifocality on patient survival is controversial; however, each nodule should be evaluated for an accurate prognosis.

**Table 1:** Patient characteristics of ipsilateral multiple renal masses

Case	Age	Sex	Location	Tumor Size (cm)	TNM	Grade	Histologic subtypes
1	64	M	Right	4.0	pT1aN0M0	2	papillary RCC, type I / metanephric adenoma
2	53	M	Right	5.3	pT1bN0M0	2	clear cell RCC/ clear cell RCC
3	66	F	Right	2.5	pT1aN0M0	2	Clear cell RCC/ clear cell RCC
4	65	M	Left	4.6	pT3aN0M0	2	Clear cell RCC/ clear cell RCC
5	57	M	Right	2.5	pT1aN0M0	3	Clear cell RCC/ clear cell RCC
6	77	M	Right	4.9	pT1bN0M0	3	Clear cell RCC/ clear cell RCC
7	62	M	Left	5.6	pT3aN0M0	3	Clear cell RCC/ Papillary RCC, type II

In summary, we report the first unusual case of unilateral synchronous PRNRP with a *KRAS* mutation (c.35G > T/p.G12V in exon 2) and clear cell RCC with a *PIK3CA* mutation (c.1624G > A/p.E542K in exon 10).

## Data Availability

The dataset supporting the conclusions of this article is included within the article.

## Abbreviations

NGS: Next-generation sequencing
RCC: Renal cell carcinoma
ISUP: International Society of Urological Pathology
PRNRP: Papillary renal neoplasm with reverse polarity
HMW: High-molecular-weight
H&E: Hematoxylin and eosin
IGV: Integrative Genomics Viewer
